# Endocrine and Metabolic Responses to Endurance Exercise Under Hot and Hypoxic Conditions

**DOI:** 10.3389/fphys.2020.00932

**Published:** 2020-08-19

**Authors:** Haruka Yatsutani, Hisashi Mori, Hiroto Ito, Nanako Hayashi, Olivier Girard, Kazushige Goto

**Affiliations:** ^1^Graduate School of Sport and Health Science, Ritsumeikan University, Kusatsu, Japan; ^2^School of Human Science and Environment, University of Hyogo, Kobe, Japan; ^3^School of Human Sciences (Exercise and Sport Science), The University of Western Australia, Perth, WA, Australia

**Keywords:** hypoxia, hot, erythropoietin, endurance exercise, muscle oxygenation

## Abstract

**Purpose:**

We explored the effect of heat stress during an acute endurance exercise session in hypoxia on endocrine and metabolic responses.

**Methods:**

A total of 12 healthy males cycled at a constant workload (60% of the power output associated with their maximal oxygen uptake under each respective condition) for 60 min in three different environments: exercise under hot and hypoxia (H+H; fraction of inspiratory oxygen or FiO_2_: 14.5%, 32°C), exercise under hypoxia (HYP; FiO_2_: 14.5%, 23°C), and exercise under normoxia (NOR; FiO_2_: 20.9%, 23°C). After completing the exercise, participants remained in the chamber for 3 h to evaluate metabolic and endocrine responses under each environment. Changes in muscle oxygenation (only during exercise), blood variables, arterial oxygen saturation, and muscle temperature were determined up to 3 h after exercise.

**Results:**

Serum erythropoietin (EPO) level was increased to similar levels in both H+H and HYP at 3 h after exercise compared with before exercise (*P* < 0.05), whereas no significant increase was found under NOR. No significant difference between H+H and HYP was observed in the serum EPO level, blood lactate level, or muscle oxygenation at any time (*P* > 0.05). Exercise-induced serum growth hormone (GH) elevation was significantly greater in H+H compared with HYP (*P* < 0.05) and HYP showed significantly lower value than NOR (*P* < 0.05). Arterial oxygen saturation during exercise was significantly lower in H+H and HYP compared with NOR (*P* < 0.05). Furthermore, H+H showed higher value compared with HYP (*P* < 0.05).

**Conclusion:**

The serum EPO level increased significantly with endurance exercise in hypoxia. However, the addition of heat stress during endurance exercise in hypoxia did not augment the EPO response up to 3 h after completion of exercise. Exercise-induced GH elevation was significantly augmented when the hot exposure was combined during endurance exercise in hypoxia. Muscle oxygenation levels during endurance exercise did not differ significantly among the conditions. These findings suggest that combined hot and hypoxic stresses during endurance exercise caused some modifications of metabolic and endocrine regulations compared with the same exercise in hypoxia.

## Introduction

Hypoxic training (i.e., endurance training under normobaric hypoxia) is commonly used by endurance athletes to improve their endurance capacity (Vogt et al., 2001; Dufour et al., 2006). One of the key physiological mechanisms for improved endurance capacity is thought to be hypoxia-induced erythropoiesis, which may lead to improved maximal oxygen uptake (V̇O_2_max) (Levin, 2002; Millet et al., 2010). Erythropoietin (EPO) is an erythropoietic hormone derived from kidney, and endurance exercise in hypoxia increases EPO production. The increased EPO contributes to augmented red blood cells (Haase, 2013; Turner et al., 2017). The hypoxia-induced increase in hemoglobin (Hb) mass increases the oxygen supply capacity for working muscle, thereby improving endurance capacity (Mairbäurl, 2013).

Many reports have shown a significant EPO response to acute endurance exercise in hypoxia (Schmidt et al., 1991; Mackenzie et al., 2008; Wahl et al., 2013). For example, Wahl et al. (2013) found that endurance exercise (cycling) for 90 min under both moderate [fraction of inspiratory oxygen (FiO_2_): 15.9%] and severe (FiO_2_: 13.2%) hypoxia significantly increased the serum EPO level 3 h after completion of exercise. Similarly, Mackenzie et al. (2008) showed that 90 min of rest under hypoxia (FiO_2_: 14.8%) followed by running for 30 min at 50% of V̇O_2__max_ significantly increased the serum EPO level.

Aside from hypoxia, heat stress during endurance exercise augments hematological variables (Lee et al., 2016). Hot exposure during an acute endurance exercise session decreased endurance performance due to disrupted homeostasis (e.g., a greater increase in core temperature and augmented dehydration) (Cheuvront et al., 2003; Périard et al., 2011). However, it may also increase plasma volume (PV) on the following day after completing the endurance exercise session under hot conditions (Nielsen et al., 1993; Lorenzo et al., 2010). Additionally, endurance exercise under hot condition may upregulate EPO production. Although the effect of acute endurance exercise under hot condition on EPO production remains unclear, 10 consecutive days of endurance training at 40°C (90 min of cycling/session) significantly increased the serum EPO level (compared with the baseline level before commencing intervention), and additional hypoxic exposure at night (8 h/day) did not cause any further increase (Rendell et al., 2017). Currently, exercise-induced EPO responses to a combination of hypoxic and heat stressors during acute endurance exercise have not been explored.

Therefore, the purpose of the present study was to compare endocrine (in particular the EPO) and metabolic responses to submaximal constant-intensity cycling exercise under (1) hot and hypoxia, (2) hypoxia, and (3) normoxia. We hypothesized that exercise-induced EPO production would be augmented when hot and hypoxia were combined.

## Materials and Methods

### Participants

A total of 12 healthy physically active men [mean ± standard error (SE) age, height, and body weight were 21.5 ± 0.3 years, 168.1 ± 1.2 cm, and 63.6 ± 2.0 kg, respectively] volunteered for this study. All participants were born and living near sea level. They were informed of the experimental procedures and possible risks associated with this study before they provided written consent. The present study was approved by the Ethics Committee for Human Experiments at Ritsumeikan University, Japan.

### Experimental Overview

All participants visited the laboratory on six occasions. During the initial three visits, they completed an incremental pedaling test on a cycle ergometer (Aerobike 75XLIII; Konami Corporation, Tokyo, Japan) under either hot and hypoxia (H+H; FiO_2_: 14.5%, 32°C), normobaric hypoxia (HYP; F_i_O_2_: 14.5%, 23°C), or normoxia (NOR; F_i_O_2_: 20.9%, 23°C) to evaluate V̇O_2__max_. The order of each condition was randomized, and each test was performed at least 2 days apart. The test began at 50 W, and the load was progressively increased by 30 W every 2 min. Exercise was terminated when the participant remained below 70 rpm for >5 s (exhaustion). Respiratory gases were collected and analyzed using an automatic gas analyzer (AE300S; Minato Medical Science Co., Ltd., Tokyo, Japan). The data collected were averaged every 30 s. The highest V̇O_2_ during exercise was defined as the V̇O_2max_.

The three main experimental conditions (visits 4–6) involved cycling for 60 min at 60% of V̇O_2__max_ under each condition (H+H, HYP, and NOR). The order of each condition was randomized, and each condition was conducted at least 5 days apart. All conditions were completed in an environmental chamber (14.8 m^2^), and hypoxic exposure was simulated by increasing the nitrogen level in the room (Morishima et al., 2014; Kasai et al., 2017). Venous blood samples were collected before exercise, 20, 40, and 60 min after commencing exercise, and 3 h after completing exercise to evaluate the serum EPO and growth hormone (GH) levels, blood lactate, glucose, Hb levels, hematocrit (Hct) value, partial pressure of oxygen (pO_2_), partial pressure of carbon dioxide (pCO_2_), pH, and bicarbonate iron (HCO_3_^–^) levels.

### Experimental Conditions

Exercise (Aerobike 75XLIII; Konami Corporation, Tokyo, Japan) was commenced following 30 min of rest after entering the chamber and obtaining baseline measurements. After completing the exercise, participants remained in the chamber for 3 h to evaluate metabolic and endocrine responses during the post-exercise period. The total duration of each session was approximately 4.5 h (including rest for 30 min, exercise for 60 min, and rest for 3 h post-exercise).

During all conditions, the FiO_2_ level was not displayed to the participant. Water intake (2 ml/body weight) was allowed before exercise and at 20 and 40 min during exercise. During the 3 h post-exercise period, participants consumed at least 600 ml water, and this volume was matched among the three conditions.

### Measurements

#### SpO_2_ and Heart Rate (HR)

Arterial oxygen saturation (SpO_2_) was recorded continuously (every 1 s) using a finger pulse oximeter (PULSOX-Me300; Teijin Pharma Ltd., Tokyo, Japan). HR was recorded continuously during exercise (every 5 s) and at 3 h post-exercise using a wireless HR monitor (RCX5; Polar Electro, Tokyo, Japan). SpO_2_ and HR values were averaged every 10 min during exercise and every 30 min during the post-exercise period.

#### Muscle and Skin Temperatures

*Vastus lateralis* muscle and skin temperatures were measured continuously (at a distance of 50% between the greater trochanter and lateral condyle of the femur) before (after entering the chamber) and during exercise and at 3 h post-exercise. Muscle temperature was evaluated non-invasively (every 2 s) using a probe-type thermometer (Core temp CM-210; TERMO Co., Ltd., Tokyo, Japan) (Wakabayashi et al., 2018). Skin temperature was monitored (every 1 s) using a probe-type thermometer and logger (NT Logger N543; Nikkiso-Therm Co., Ltd., Tokyo, Japan) (Maruyama et al., 2019). Muscle and skin temperatures were averaged every 10 min during exercise and every 30 min during the post-exercise period.

#### Blood Variables

Following an overnight fast, the participants arrived at the laboratory in the morning (8:00 am). After 30 min of rest, baseline blood samples were collected from an inserted cannula in the antecubital vein. Afterward, blood samples were further collected at 20, 40, and 60 min after commencing exercise and at 3 h after completing the exercise. To obtain serum, the blood samples were centrifuged for 10 min at 4°C (3000 rpm) and stored at −80°C prior to analysis. Serum EPO and GH levels were measured in a clinical laboratory (SRL Inc., Tokyo, Japan) using an atomic absorption method (for EPO) and radioimmunoassay (for GH). The intra-assay coefficients of variation were 7.2% for EPO and 3.5% for GH, respectively.

Whole blood samples were also used to determine the blood Hb level, Hct value, pO_2_, pCO_2_, pH, and HCO_3_^–^ levels using a blood gas analyzer (OPTI CCA-TS2; Sysmex Corporation, Hyogo, Japan). Exercise-induced changes in PV were calculated using the Hb level and Hct value, as described previously (Dill and Costill, 1974).

Blood glucose and lactate levels were measured every 20 min during exercise and at 3 h after completing the exercise using a glucose analyzer (Freestyle; Nipro Co., Osaka, Japan) and lactate analyzer (Lactate Pro; Arkray Inc., Kyoto, Japan), respectively.

#### Muscle Oxygenation Evaluated Using Near-Infrared Spectroscopy (NIRS)

Before exercise (before and after entering the chamber) and 20, 40, and 60 min after commencing exercise, the oxygenated Hb (oxy-Hb), deoxygenated Hb (deoxy-Hb), total Hb (total-Hb), and tissue oxygen saturation (StO_2_) were recorded from the *vastus lateralis* muscle (at a distance of 50% from the greater trochanter and lateral condyle of the femur) using NIRS (Hb14; ASTEM Co., Ltd., Kanagawa, Japan) (Yamaguchi et al., 2019). To determine muscle oxygenation variables, the NIRS probe was placed on the muscle at an inter-optode distance of 30 mm. All signals were recorded at a sampling frequency of 10 Hz. NIRS data during exercise were expressed relative to the baseline value (data collected before entering the chamber), which was determined under normoxic (FiO_2_: 20.9%) and normal (23°C) conditions.

#### Respiratory Gas Parameters

Respiratory gas parameters were collected every 20 min during exercise (15–20, 35–40, and 55–60 min during the 60-min exercise period). Oxygen uptake (V̇O_2_), carbon dioxide output (V̇CO_2_), and minute ventilation (V̇E) were evaluated using an automatic gas analyzer (AE300S; Minato Medical Science Co., Ltd., Tokyo, Japan). The respiratory exchange ratio (RER) was calculated using the V̇O_2_ and V̇CO_2_ values. All respiratory values were averaged every 30 s.

### Statistical Analysis

All data are presented as means ± SE. Two-way repeated-measures analysis of variance (ANOVA) was used to investigate the main effects of condition, time, and the interaction between condition and time. When a significant effect was found, the Tukey–Kramer *post-hoc* test was performed. Absolute workload, average SpO_2_, HR, muscle temperature, and skin temperature were compared using one-way ANOVA followed by a *post-hoc* test. For all tests, a *P*-value < 0.05 was considered significant. The values of partial eta squared (partial η^2^) for one-way and two-way repeated-measures ANOVA were calculated to present effect size. The partial η^2^ around of around 0.02, 0.13, and 0.26 were considered as “small,” “medium,” and “large” (Dalton et al., 2017).

## Results

### Absolute Workload During 60 min of Endurance Exercise

Absolute workload during exercise under each condition was significantly lower in H+H (110 ± 4 W) and HYP (107 ± 5 W) than in NOR (136 ± 4 W).

### Serum EPO Level

**FIGURE 1:**
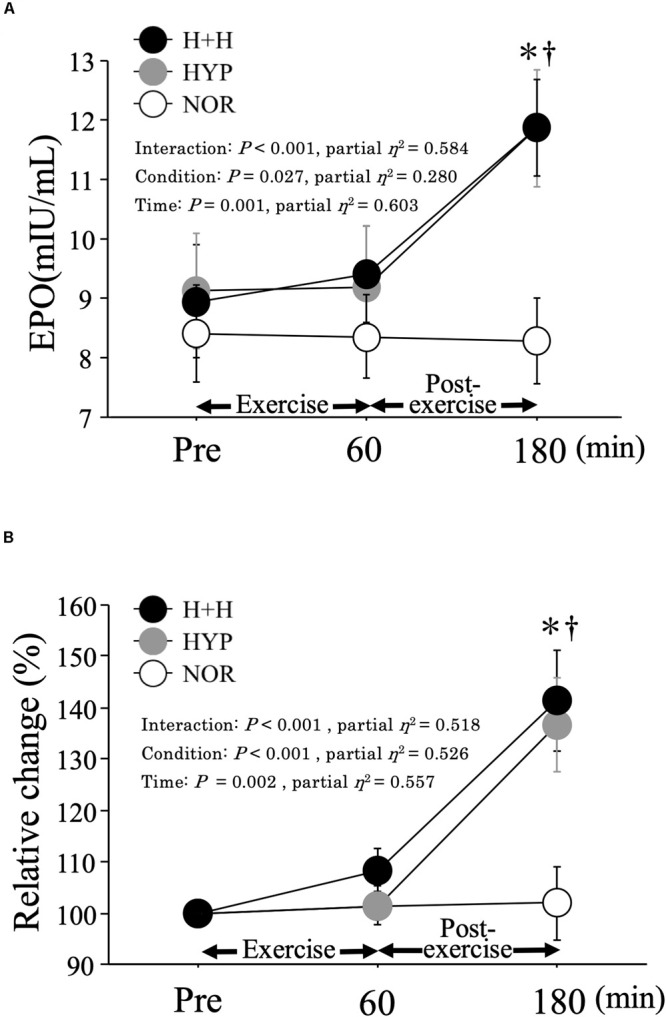
Absolute **(A)** and relative **(B)** changes in serum EPO level during exercise and post-exercise. Values are means ± SE. ^∗^*P* < 0.05 vs. Pre. ^†^ *P* < 0.05 vs. NOR.

Figure 1 presents the absolute (A) and relative (B) changes in the serum EPO level before and after exercise. In both H+H and HYP, the serum EPO level was significantly increased at 3 h after exercise and was significantly higher than that in NOR. However, no significant difference in the serum EPO level was observed between the H+H and HYP. The relative change in the EPO level at 3 h after exercise was significantly higher in both H+H (141.4 ± 9.9%) and HYP (136.7 ± 9.1%) than in NOR (101.9 ± 7.1%), but no significant difference was observed between H+H and HYP.

### SpO_2_ and HR

**FIGURE 2:**
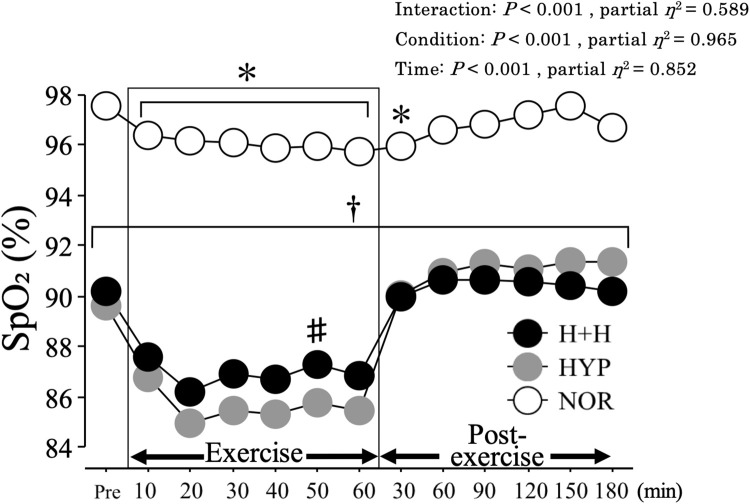
SpO_2_ during exercise and post-exercise. Values are means ± SE. ^∗^*P* < 0.05 vs. Pre. ^†^*P* < 0.05 vs. NOR. ^#^*P* < 0.05 vs. HYP. *Shadow box indicates exercise duration.*

Figure 2 presents the changes in the SpO_2_ level from before to after exercise. The average SpO_2_ during exercise was significantly lower in H+H (86.9 ± 0.6%) and HYP (85.5 ± 0.6%) than in NOR (96.1 ± 0.3%, *P* < 0.01, partial η^2^ = 0.95). The SpO_2_ level during exercise was significantly higher in H+H than in HYP at the 50-min time point. During the 3 h post-exercise period, the average SpO_2_ remained significantly lower in both H+H (90.4 ± 0.4%) and HYP (91.0 ± 0.5%) than in NOR (96.8 ± 0.2%, *P* < 0.01, partial *η^2^* = 0.97). The average HR during exercise was significantly higher in H+H (152 ± 3 bpm) and NOR (149 ± 3 bpm) than in HYP (142 ± 4 bpm, *P* = 0.01, partial η^2^ = 0.37). During the post-exercise period, the average HR was significantly higher in H+H (92 ± 3 bpm) compared with HYP (78 ± 3 bpm) and NOR (78 ± 3 bpm, *P* < 0.01, partial η^2^ = 0.74).

### Muscle and Skin Temperatures

**FIGURE 3:**
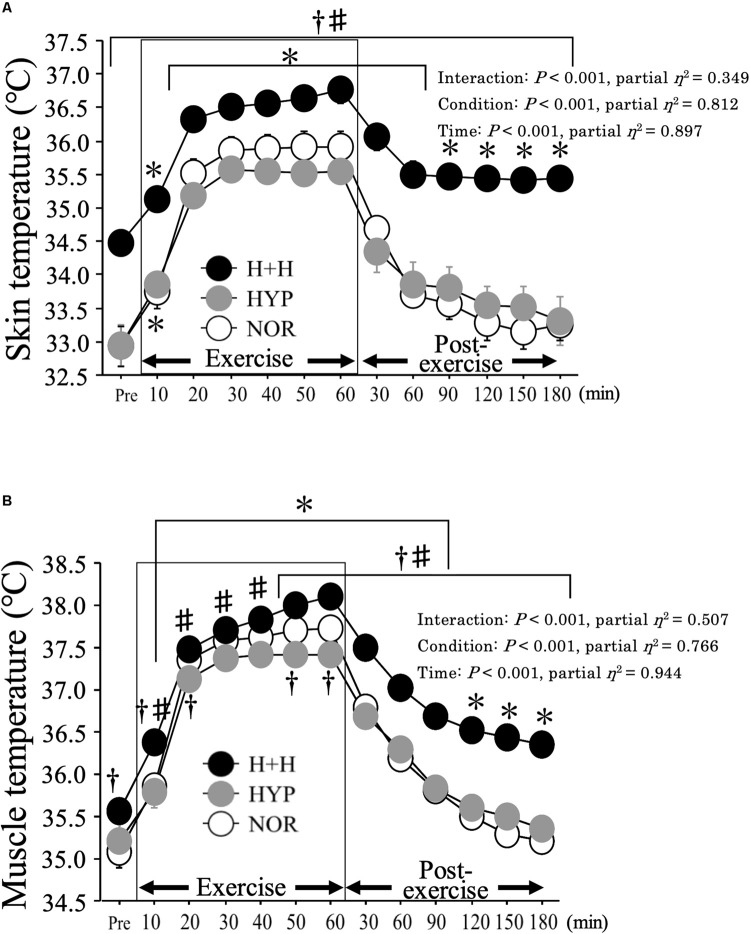
Skin **(A)** and muscle **(B)** temperatures during exercise and post-exercise. Values are means ± SE. ^∗^*P* < 0.05 vs. Pre. ^†^*P* < 0.05 vs. NOR. ^#^*P* < 0.05 vs. HYP. *Shadow box indicates exercise duration.*

Figure 3 presents the skin (A) and muscle (B) temperatures before and after exercise. Muscle temperature increased significantly during exercise under all conditions, with a higher average value in H+H (37.6 ± 0.1°C) than in HYP (37.1 ± 0.1°C) and NOR (37.3 ± 0.1°C, *P* < 0.01, partial η^2^ = 0.94). During the post-exercise period, the average muscle temperature was significantly higher in H+H (36.8 ± 0.1°C) than in HYP (35.9 ± 0.1°C) and NOR (35.8 ± 0.1°C, *P* < 0.01, partial η^2^ = 0.85).

Skin temperature increased during exercise, with a significantly higher average value in H+H (36.3 ± 0.2°C) than in HYP (35.2 ± 0.1°C) and NOR (35.5 ± 0.2°C, *P* < 0.01, partial η^2^ = 0.61). During the post-exercise period, the average skin temperature remained significantly elevated in H+H (35.6 ± 0.2°C) compared with HYP (33.7 ± 0.3°C) and NOR (33.6 ± 0.2°C, *P* < 0.01, partial η^2^ = 0.82).

### Blood Variables

Table 1 presents the blood pO_2_, pCO_2_, pH, and HCO_3_^–^ levels. The blood pO_2_ level during exercise was significantly higher in NOR than in HYP and H+H. The blood pCO_2_ level during exercise and post-exercise was significantly lower in H+H than in HYP and NOR. In contrast, the blood pH levels were significantly higher in H+H than in NOR and HYP. The HCO_3_^–^ level decreased significantly during exercise, and it was significantly lower in H+H than in HYP and NOR.

**TABLE 1:** Blood pO_2_, pCO_2_, pH, and HCO_3_^–^ levels.

		**Pre**	**20 min**	**40 min**	**60 min**	**Post 180 min**	**ANOVA (partial η^2^)**		
							**Interaction**	**Condition**	**Time**
PO2 (kPa)	H+H	7.07 ± 0.3	7.03 ± 0.2^†^	7.01 ± 0.1^†^	7.06 ± 0.2^†^	6.55 ± 0.4^†^	<0.001 (0.679)	<0.001 (0.746)	<0.001 (0.859)
	HYP	5.99 ± 0.4	6.35 ± 0.2^†^	6.66 ± 0.1^†^	6.44 ± 0.1^†^	3.53 ± 0.2*			
	NOR	6.81 ± 0.7	9.92 ± 0.5*	10.04 ± 0.5*	9.77 ± 0.4*	4.11 ± 0.4*			
PCO2 (kPa)	H+H	5.46 ± 0.1^†^	4.96 ± 0.1^†^	4.76 ± 0.1*^†^	4.81 ± 0.2*	5.18 ± 0.1^†^	<0.001 (0.445)	<0.001 (0.696)	<0.001 (0.823)
	HYP	5.82 ± 0.2	5.25 ± 0.1*	5.01 ± 0.1*^†^	5.04 ± 0.1*	6.66 ± 0.1*			
	NOR	6.15 ± 0.2	5.42 ± 0.1*	5.27 ± 0.1*	5.21 ± 0.1*	6.64 ± 0.2*			
	H+H	7.42 ± 0.01^†^	7.42 ± 0.01^†^	7.44 ± 0.01^†^	7.45 ± 0.01*^†^	7.43 ± 0.01^†^	0.001 (0.348)	<0.001 (0.809)	<0.001 (0.705)
pH	HYP	7.40 ± 0.01	7.40 ± 0.01	7.42 ± 0.01^†^	7.44 ± 0.01*	7.37 ± 0.01*			
	NOR	7.39 ± 0.01	7.39 ± 0.01	7.41 ± 0.01	7.42 ± 0.01*	7.37 ± 0.01			
HCO3^–^ (mmol/L)	H+H	25.7 ± 0.4	23.5 ± 0.5*	23.5 ± 0.5*^†^	23.2 ± 0.3*^†^	25.5 ± 0.4^†^	0.010 (0.261)	0.002 (0.441)	<0.001 (0.778)
	HYP	26.5 ± 0.5	23.9 ± 0.4*	24.0 ± 0.4*	24.6 ± 0.4*	28.3 ± 0.3*			
	NOR	27.2 ± 0.4	24.0 ± 0.5*	24.6 ± 0.5*	25.0 ± 0.5*	27.9 ± 0.6			

*Values are means ± SE. ^∗^*P* < 0.05 vs. Pre. ^†^*P* < 0.05 vs. NOR. ^#^*P* < 0.05 vs. HYP. pO_2_, partial pressure of oxygen; pCO_2_, partial pressure of carbon dioxide; HCO_3_^–^, bicarbonate iron.*

Table 2 presents the blood glucose, lactate and Hb levels, and Hct value and PV. The blood glucose level decreased significantly during exercise, but there was no significant difference among the three condition. The blood lactate level significantly increased during exercise. However, these values did not significantly differ among the three condition.

**TABLE 2:** Blood glucose, lactate and Hb levels, Hct value and ΔPV.

		**Pre**	**20 min**	**40 min**	**60 min**	**Post 180 min**	**ANOVA (partial η^2^)**		
							**Interaction**	**Condition**	**Time**
Glucose (mg/dL)	H+H	911	841*	841*	862	862	0.162 (0.135)	0.055 (0.232)	<0.001 (0.483)
	HYP	892	852	842	832*	852			
	NOR	882	782*	822*	792*	813*			
Lactate (mmol/L)	H+H	1.20.1	3.80.5*	3.40.5*	3.00.4*	1.20.1	0.022 (0.256)	0.350 (0.091)	0.001 (0.627)
	HYP	1.40.1	3.30.5*	3.10.5*	2.60.4*	1.70.1			
	NOR	1.30.1	3.40.4*	2.80.5*	2.50.4*	1.40.1			
Hb (g/dL)	H+H	14.50.3	15.50.2*	15.70.2*	15.70.2*	14.80.2*^†^	0.079 (0.144)	0.028 (0.277)	<0.001 (0.842)
	HYP	14.80.2	15.80.3*	15.80.3*	15.90.3*	15.50.2*			
	NOR	14.90.3	15.70.3*	15.80.3*	15.80.3*	15.30.3*			
Hct (%)	H+H	43.40.8	46.60.7*	47.10.7*	47.20.6*	44.50.6*^†^	0.069 (0.147)	0.036 (0.260)	<0.001 (0.842)
	HYP	44.50.7	47.50.8*	47.50.8*	47.70.8*	46.60.6*			
	NOR	44.60.8	47.10.9*	47.40.9*	47.50.8*	46.00.9			
ΔPV (%)	H+H	0	−11.81.4*	−13.61.0*	−13.91.2*	−4.11.5*	0.062 (0.151)	0.516 (0.058)	<0.001 (0.848)
	HYP	0	−11.21.2*	−11.31.5*	−12.31.1*	−7.81.4*			
	NOR	0	−9.31.8*	−10.71.8*	−10.82.0*	−5.22.2			

*Values are means ± SE. ^∗^*P* < 0.05 vs. Pre. ^†^*P* < 0.05 vs. NOR. ^#^*P* < 0.05 vs. HYP. Hb, hemoglobin; Hct, hematocrit; ΔPV, percentage change in plasma volume (the values were expressed in refer to pre-exercise value).*

The blood Hb level significantly increased during exercise and was significantly lower in H+H than in HYP and NOR at 3 h after completing exercise. The blood Hct value significantly increased under all conditions, and it was significantly lower in H+H than in HYP and NOR at 3 h after completing exercise. PV significantly decreased during exercise, with no significant difference among the three conditions.

### Serum GH

**FIGURE 4:**
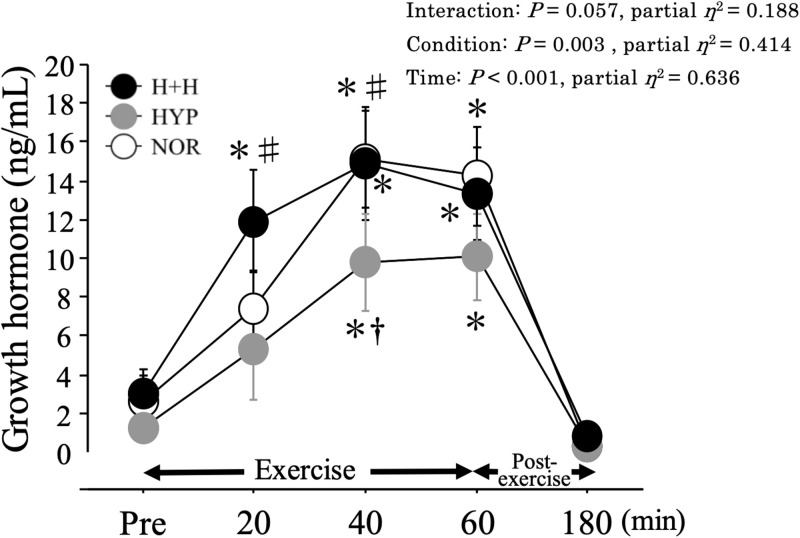
Serum growth hormone level during exercise and post-exercise. Values are means ± SE. ^∗^*P* < 0.05 vs. Pre. ^†^*P* < 0.05 vs. NOR. ^#^*P* < 0.05 vs. HYP.

Figure 4 shows the changes in serum GH level. During 60 min of exercise, the serum GH level increased significantly during exercise, with higher values in H+H and NOR than in HYP.

### Muscle Oxygenation

**FIGURE 5:**
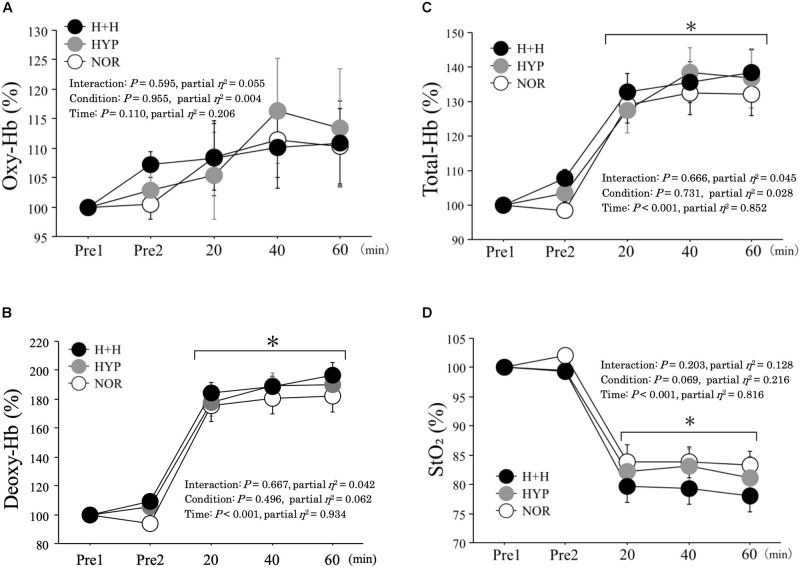
Oxy-hemoglobin **(A)**, deoxy-hemoglobin **(B)**, total-hemoglobin **(C)**, tissue oxygen saturation **(D)** during exercise. *All values were expressed as relative value (values were expressed in refer to pre-exercise value). Pre1 indicates rest under normoxic condition (normal temperature). Pre2 indicates rest under each condition.* Values are means ± SE. ^∗^*P* < 0.05 vs. Pre1.

Figure 5 presents the changes in muscle oxygenation during exercise. The oxy-Hb level during exercise did not differ among the three condition (NOR; 110.3 ± 6.3%, HYP; 113.5 ± 10.0%, H+H; 110.8 ± 7.2% 60 min during exercise). Moreover, the deoxy-Hb level increased during exercise under all conditions, with no significant difference among three conditions (NOR; 182.0 ± 10.8%, HYP; 190.2 ± 10.2%, H+H; 196.7 ± 8.6% 60 min during exercise). The total-Hb level significantly increased during exercise in all conditions, but no significant difference was observed among the three conditions (NOR; 132.3 ± 6.4%, HYP; 136.8 ± 8.5%, H+H; 138.5 ± 6.5% 60 min during exercise). The StO_2_ decreased significantly during exercise, whereas no significant difference was observed among the three conditions (NOR; 83.2 ± 2.5%, HYP; 81.2 ± 3.6%, H+H; 78.0 ± 2.7% 60 min during exercise).

### Respiratory Gas Parameters During Exercise

Table 3 presents the changes in respiratory gas parameters during exercise. V̇O_2_ and V̇CO_2_ values were significantly lower in H+H and HYP than in NOR at all time points. RER was significantly higher in H+H and HYP than in NOR at all time points. In terms of V̇E, it was significantly lower in HYP compared with NOR.

**TABLE 3:** Respiratory gas parameters during exercise.

		**20 min**	**40 min**	**60 min**	**ANOVA (partial η^2^)**		
					**Interaction**	**Condition**	**Time**
V̇O^2^ (mL/min)	H+H	1661 ± 58^†^	1720 ± 61^†^	1783 ± 65^†^	0.208 (0.122)	<0.001 (0.745)	<0.001 (0.914)
	HYP	1604 ± 59^†^	1652 ± 66^†^	1693 ± 67^†^			
	NOR	2006 ± 57	2068 ± 59	2145 ± 55			
V̇CO^2^ (mL/min)	H+H	1573 ± 55^†^	1580 ± 55^†^	1623 ± 61^†^	0.405 (0.085)	<0.001 (0.639)	0.008 (0.357)
	HYP	1502 ± 56^†^	1508 ± 63^†^	1516 ± 62^†^			
	NOR	1808 ± 54	1812 ± 54	1846 ± 50			
V̇E (L/min)	H+H	53.0 ± 2.2	56.6 ± 2.5	61.8 ± 3.2	0.149 (0.140)	0.037 (0.304)	<0.001 (0.722)
	HYP	49.9 ± 2.2^†^	53.0 ± 2.7^†^	55.4 ± 3.1^†^			
	NOR	56.5 ± 2.1	59.0 ± 2.6	62.9 ± 3.3			
RER	H+H	0.95 ± 0.01^†^	0.92 ± 0.01^†^	0.91 ± 0.01^†^	0.577 (0.062)	<0.001 (0.539)	<0.001 (0.855)
	HYP	0.94 ± 0.01^†^	0.91 ± 0.01^†^	0.90 ± 0.01^†^			
	NOR	0.90 ± 0.01	0.88 ± 0.01	0.86 ± 0.01			

*Values are means ± SE. ^†^*P* < 0.05 vs. NOR. V̇O_2_, oxygen uptake; V̇CO_2_, carbon dioxide output; V̇E, minute ventilation; RER, respiratory exchange ratio.*

## Discussion

The main purpose of the present study was to evaluate the endocrine (in particular EPO) responses to 60 min of endurance exercise under combined hot and hypoxic condition. We found that both the H+H and HYP caused significant increases in the serum EPO level 3 h after completion of exercise, but no difference was observed between H+H and HYP. Moreover, exercise-induced GH elevation was significantly augmented when the hot exposure was combined during exercise in hypoxia. Therefore, additional heat stress during endurance exercise in hypoxia did not affect the erythropoietic response compared with the same exercise under hypoxia alone, but it may be beneficial for increasing GH response.

Endurance exercise in HYP resulted in a significantly higher serum EPO level compared with NOR at 3 h after completion of exercise. This observation is consistent with previous studies reporting a 58% increase after endurance exercise under severe hypoxia (FiO_2_: 13.2%), as well as 61% (FiO_2_: 15.9%) and 25% (FiO_2_: 14.8%) increases after endurance exercise under moderate hypoxia (Mackenzie et al., 2008; Wahl et al., 2013). However, we observed that heat stress during endurance exercise under hypoxia did not further increase the EPO response. Another unique observation in our study was that the SpO_2_ level during endurance exercise was significantly higher in H+H than in HYP, despite similar FiO_2_ (14.5%). Body temperature and V̇E were further elevated in H+H than in HYP, leading to an increased blood pO_2_ level. Abbiss et al. (2007) compared blood pH, partial pressure of arterial oxygen, and partial pressure of arterial carbon dioxide levels during prolonged self-paced cycling under different environmental temperatures (10, 22, and 34°C). They observed that hot trial (34°C) resulted in higher blood pH and lower partial pressure of arterial carbon dioxide levels compared with cooler trials (10 and 22°C). Elevated blood pH during endurance exercise in hot would reflect augmented exercise-induced respiratory alkalosis. Similarly, our H+H condition induced significantly higher blood pH with a lower pCO_2_ level compared with HYP and NOR. Overall, additional hot exposure during endurance exercise in hypoxia would be expected to attenuate the reduction in SpO_2_. Therefore, combining hot and hypoxia during endurance exercise may not augment EPO production compared with the same endurance exercise under hypoxia alone. However, in the present study, we did not prepare hot alone condition. Therefore, it remains unclear how the heat stress itself affects EPO response.

Both muscle and skin temperatures increased significantly as exercise progressed under all conditions, but these values were significantly higher in H+H than in both HYP and NOR. Heat stress was rather modest in the present study (32°C) compared with previous studies (>35°C; Hunter et al., 2002; Lorenzo et al., 2010; Guy et al., 2016). However, in H+H, muscle and skin temperatures reached 38.1 ± 0.1°C and 36.2 ± 0.2°C, respectively, by the end of exercise. These levels are comparable with those observed in previous studies, reporting skin temperatures of approximately 35°C during exercise under 35°C (Bradbury et al., 2019). We also evaluated muscle oxygenation because previous studies reported that lower muscle oxygenation (increased muscle deoxygenation) may aggravate the accumulation of exercise-induced metabolites (Grassi et al., 1999). Moreover, the total-Hb level evaluated by NIRS reflects blood volume in muscles (van Beekvelt et al., 2001; De smet et al., 2017). Considering that heat stress increases blood flow due to vasodilatation (Périard et al., 2015) we hypothesized that exercise-induced increases in total-Hb would be augmented in H+H. In contrast to our hypothesis, there was no significant difference among the three conditions in either the muscle oxygenation level or total-Hb level. As potential reasons, the absolute workload was significantly lower in both H+H (19%) and HYP (21%) compared with NOR due to a significantly lower V̇O_2__max_. Moreover, augmented skin blood flow during endurance exercise in hot condition may attenuate muscle blood flow (Périard et al., 2013) leading to the lack of increase in total-Hb in H+H.

We expected the exercise-induced GH elevation would be increased in both H+H and HYP compared with NOR because hypoxia has previously been shown to augment the exercise-induced GH response (Kon et al., 2010, 2015; Kurobe et al., 2015; Filopoulos et al., 2017). Unexpectedly, exercise-induced GH elevation was significantly lower in HYP compared with NOR due to a lower absolute workload (Felsing et al., 1992). However, H+H presented significantly greater GH elevation than HYP, suggesting the benefit of additional hot stress during endurance exercise in hypoxia.

Several limitations should be considered when interpreting our results. First, we did not implement a hot-alone condition. Therefore, it remains unclear whether heat stress alone affects the EPO response. Second, we did not measure core temperature because we used moderate heat stress (32°C). Finally, we evaluated the EPO response to endurance exercise until 3 h after exercise. However, previous studies have shown large interindividual differences in the exercise-induced EPO response (Sinex and Chapman, 2015). Thus, extending measurements during the post-exercise period to beyond 3 h may be required.

## Conclusion

The serum EPO level significantly increased with endurance exercise under hypoxic conditions. However, the addition of heat stress during endurance exercise in hypoxia did not augment the EPO response at 3 h after completion of the exercise. Exercise-induced GH elevation was significantly augmented when the hot exposure was combined during endurance exercise in hypoxia. Muscle oxygenation levels during endurance exercise did not differ significantly among the conditions. These findings suggest that combined hot and hypoxic stresses during endurance exercise caused some modifications of metabolic and endocrine regulations compared with the same exercise in hypoxia.

## Data Availability Statement

All datasets generated for this study are included in the article/supplementary material.

## Ethics Statement

The present study was approved by the Ethics Committee for Human Experiments at Ritsumeikan University, Japan. The patients/participants provided their written informed consent to participate in this study.

## Author Contributions

HY and KG designed the study. HY, HM, HI, and NH performed the material preparation, data collection, and analysis. HY, OG, and KG wrote the first draft of the manuscript. All authors read and approved the final manuscript.

## Conflict of Interest

The authors declare that the research was conducted in the absence of any commercial or financial relationships that could be construed as a potential conflict of interest.
